# Supplementary figures and images for: Landscape-scale spatial variations of pre-Columbian anthropogenic disturbances at three ring ditch sites in French Guiana
Source: PLoS One. 2024 Sep 26;19(9):e0298714. doi: 10.1371/journal.pone.0298714 (PMC11426519; doi:10.1371/journal.pone.0298714)

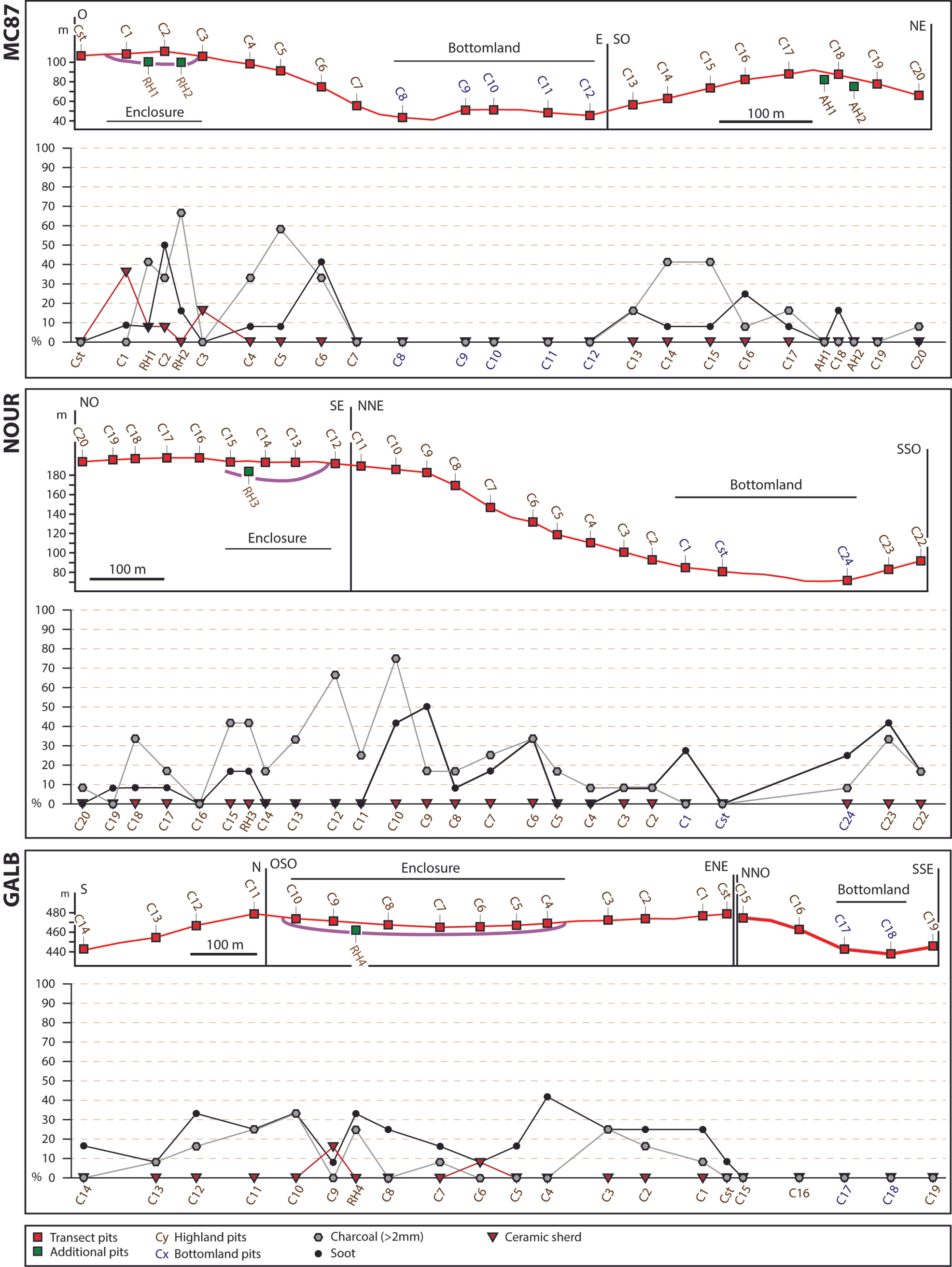

Supplement: S1 Fig — (TIF) [file pone.0298714.s001.tif]

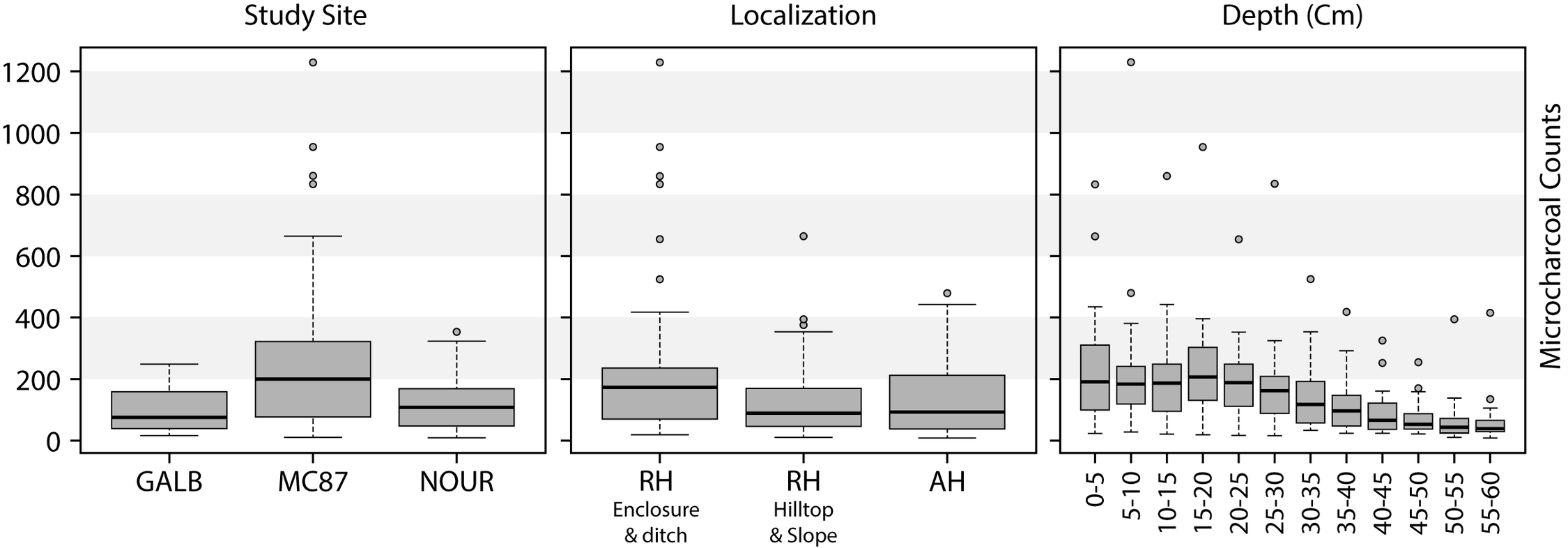

Supplement: S2 Fig — (TIF) [file pone.0298714.s002.tif]

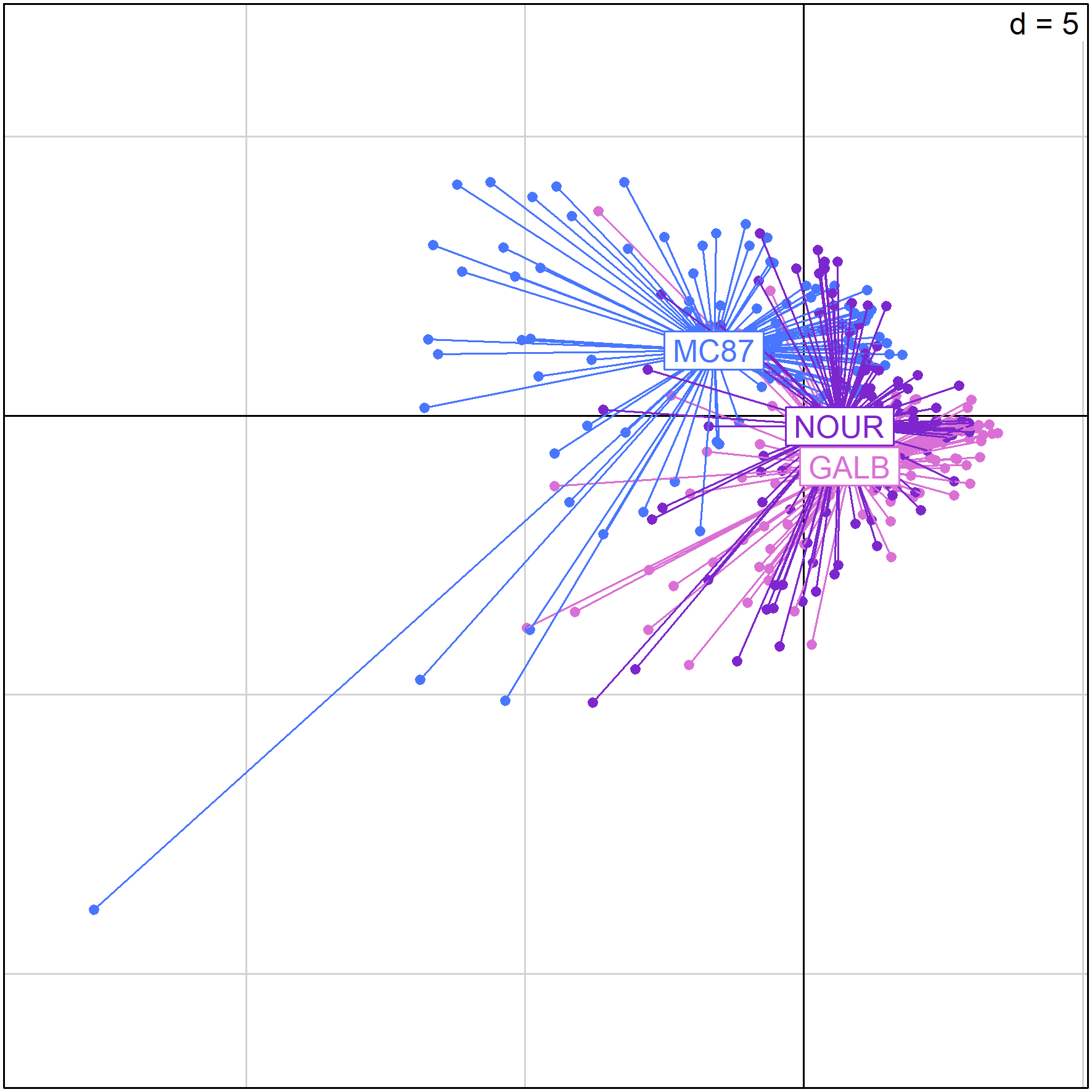

Supplement: S3 Fig — (TIF) [file pone.0298714.s003.tif]

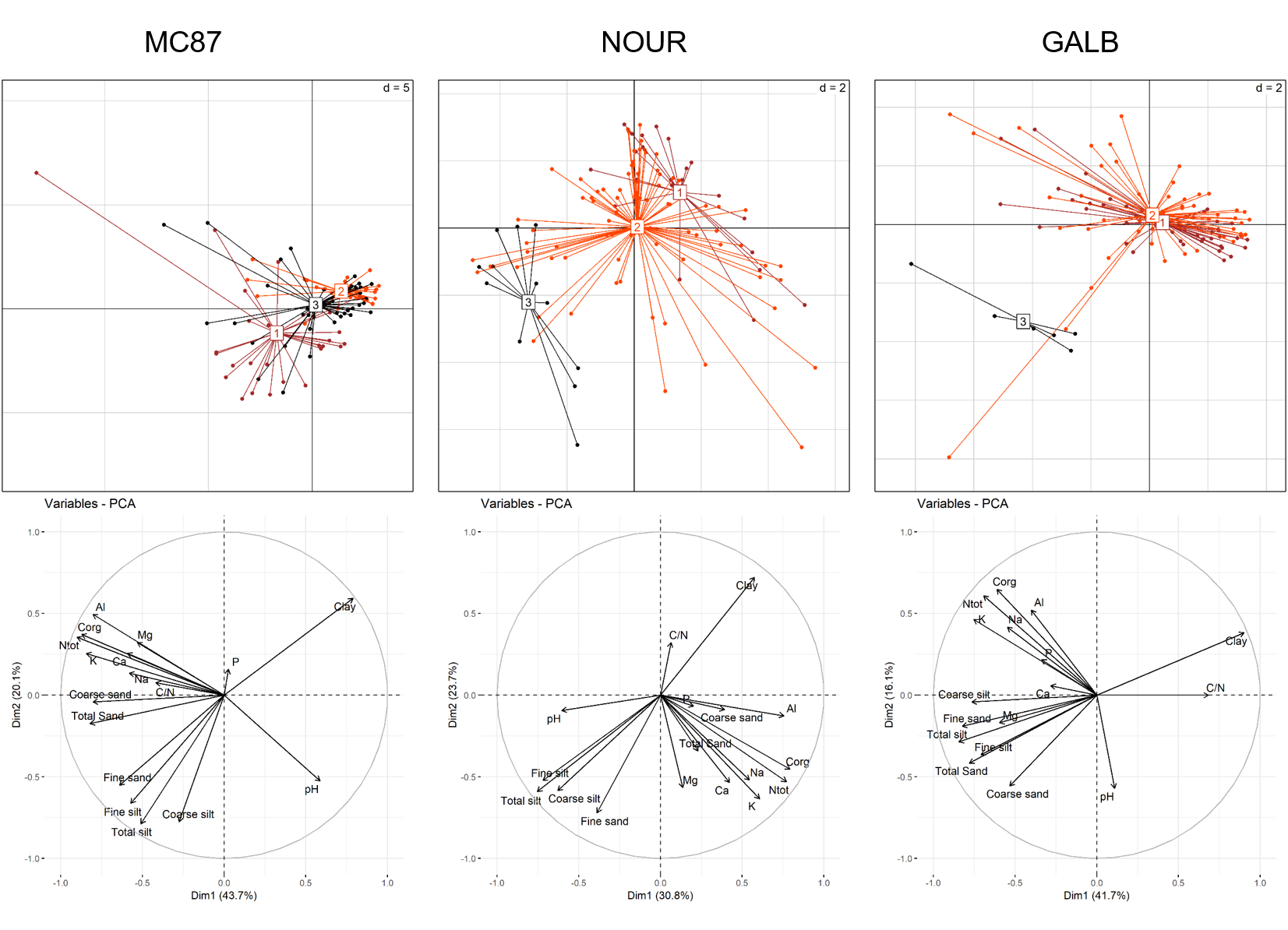

Supplement: S4 Fig — 1: Ring ditch enclosure and ditch; 2: Ring ditch plateau and slopes; 3: Adjacent hill. (TIF) [file pone.0298714.s004.tif]

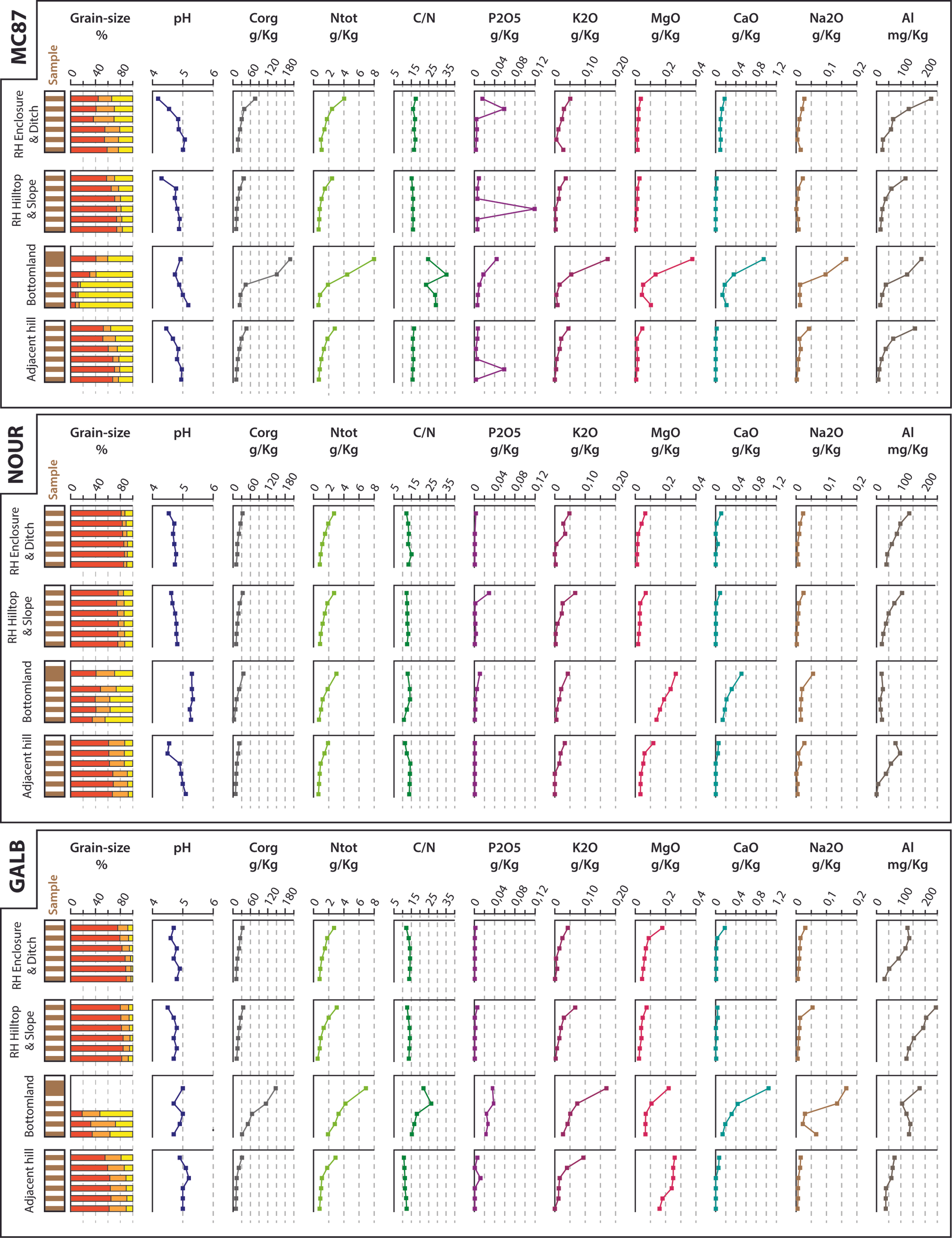

Supplement: S5 Fig — Averaged values are calculated for each depth from all pits in the same localization (Ring ditch Enclosure & Ditch, Ring ditch Hilltop & Slope, Bottomland, Adjacent hill). (TIF) [file pone.0298714.s005.tif]

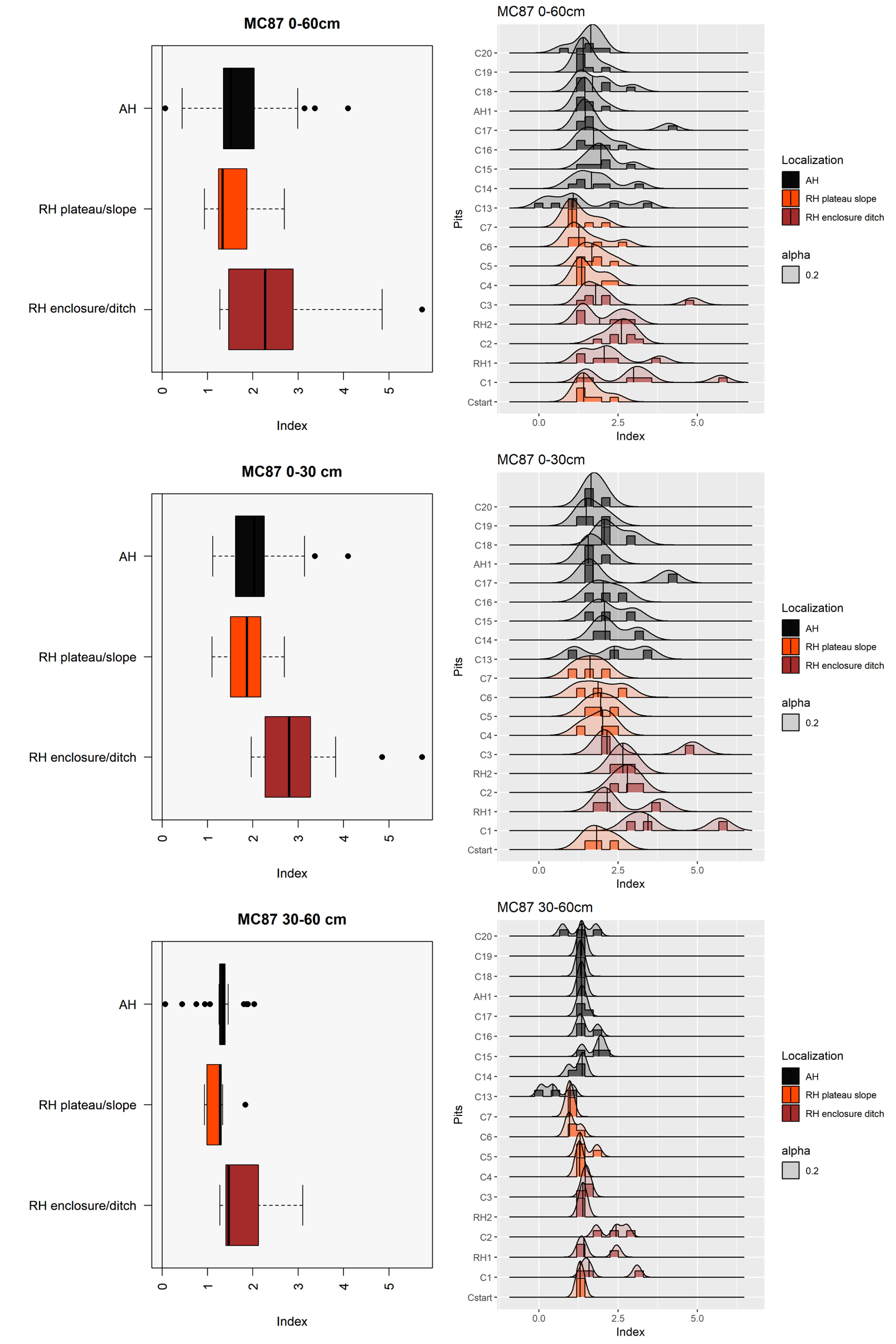

Supplement: S6 Fig — Entire soil profile (upper pane), between 0 and 30 cm deep (middle pane), between 30 and 60 cm deep (lower pane). (TIF) [file pone.0298714.s006.tif]

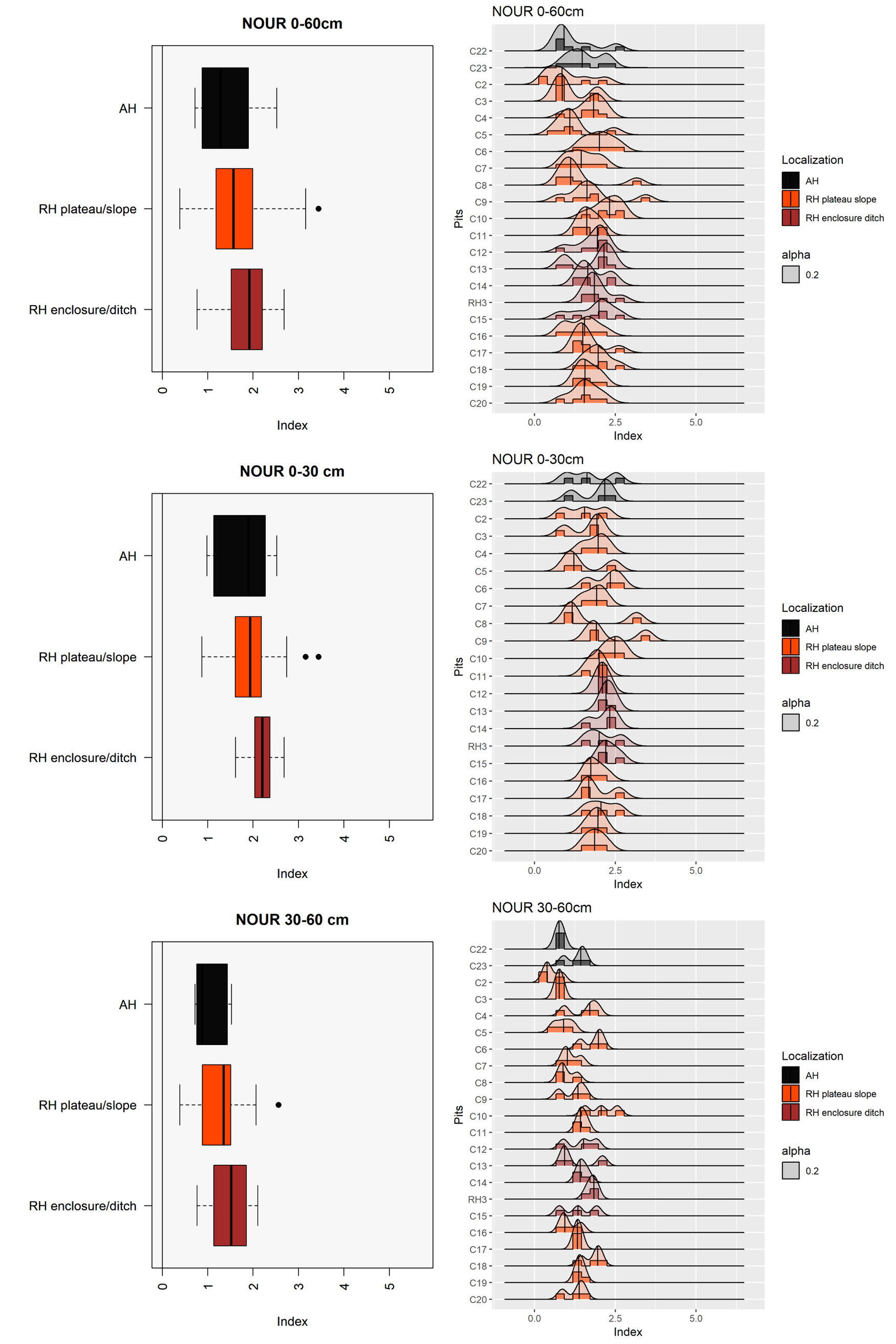

Supplement: S7 Fig — Entire soil profile (upper pane), between 0 and 30 cm deep (middle pane), between 30 and 60 cm deep (lower pane). (TIF) [file pone.0298714.s007.tif]

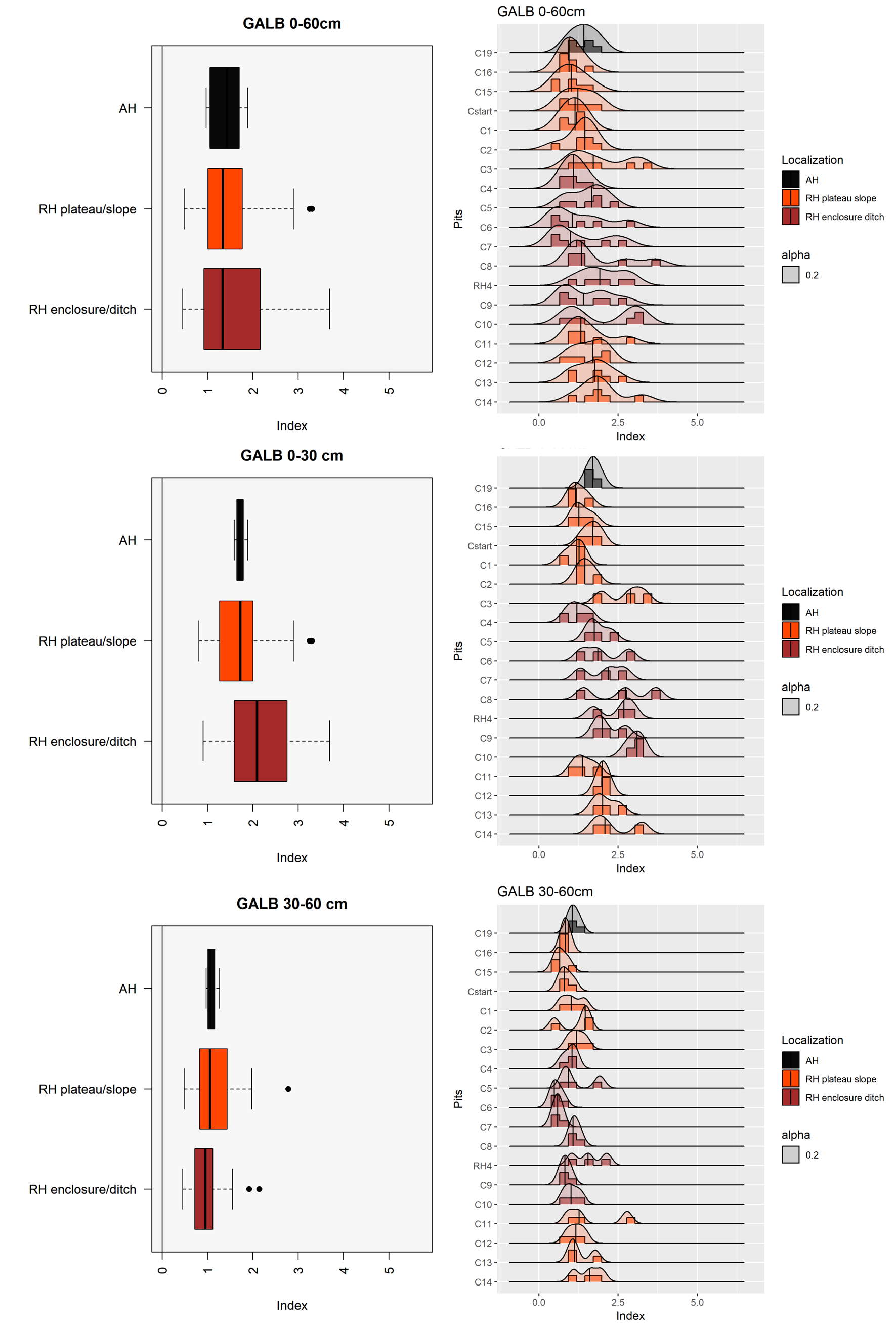

Supplement: S8 Fig — Entire soil profile (upper pane), between 0 and 30 cm deep (middle pane), between 30 and 60 cm deep (lower pane). (TIF) [file pone.0298714.s008.tif]
