# Supplementary material for: Landscape-scale spatial variations of pre-Columbian anthropogenic disturbances at three ring ditch sites in French Guiana
Source: PLoS One. 2024 Sep 26;19(9):e0298714. doi: 10.1371/journal.pone.0298714 (PMC11426519; doi:10.1371/journal.pone.0298714)
Supplement: S1 Table — (DOCX) [file pone.0298714.s009.docx]

**S1 Table. Non-exhaustive literature overview of earthwork radiocarbon dates in Amazonia**.

| **Region** | **Reference** | **Radiocarbon dates** |
| --- | --- | --- |
| North Amazonia  (Guiana shield, French Guiana) | Bodin et al. 2020 | CE 650- 950  (1300-1000 B.P.) and  CE 1350-1550 (600-400 B.P.) |
| North Amazonia  (Guiana shield, French Guiana) | Brancier et al. 2014 | CE 500 -1100 |
| South Amazonia  (Upper Tapajos, Brazil) | de Souza et al. 2018 | CE 1250-1500 |
| South Amazonia  (Upper Xingu, Brazil) | Heckenberger et al. 2003 | CE 1200-1600 |
| South-west Amazonia  (Llanos de Mojos, Bolivia) | Prümers et al. 2022 | CE 500-1400 |
| South-west Amazonia  (Llanos de Mojos, Bolivia) | Carson et al. 2015 | CE 1200-1400  (750-550 B.P.), with decline in population activity 1350-1450 (600-500 B.P.) |
| South-west Amazonia  (Llanos de Mojos, Bolivia) | Carson et al. 2014 | CE 1150-1450  (500-800 B.P.) |
| West Amazonia  (Acre, Brazil) | Pärssinen et al. 2009 | CE 1244-1378 |
| West Amazonia  (Acre, Brazil) | Schaan et al. 2012 | BCE 50 – CE 1250  (2000-700 B.P.) |
| West Amazonia  (Acre, Brazil) | Saunaluoma and Schaan 2012 | BCE 1600 - CE 1300, with the most intensive period BCE 200 - CE 900 |
