# Supplementary material for: Landscape-scale spatial variations of pre-Columbian anthropogenic disturbances at three ring ditch sites in French Guiana
Source: PLoS One. 2024 Sep 26;19(9):e0298714. doi: 10.1371/journal.pone.0298714 (PMC11426519; doi:10.1371/journal.pone.0298714)
Supplement: S2 Table — The calibration was performed using the OxCal 4.4 program with the IntCal20 calibration curve. (DOCX) [file pone.0298714.s010.docx]

**S2 Table. Radiocarbon dates obtained from the three study sites.** The calibration was performed using the OxCal 4.4 program with the IntCal20 calibration curve.

| **Study site** | **pit** | **depth** | **cal. age (BCE / CE)** |
| --- | --- | --- | --- |
| MC87 | RH1 | 0-5 | 1223 - 1280 CE (95.4%) |
| MC87 | RH1 | 10-15 | 1214 - 1278 CE (95.4%) |
| MC87 | RH2 | 25-30 | 993 - 1155 CE (95.4%) |
| MC87 | RH2 | 30-35 | 707 - 945 CE (95.4%) |
| MC87 | C2 | 15-20 | 1282 - 1395 CE (95.4%) |
| MC87 | C2 | 40-45 | 1298 - 1398 CE (95.4%) |
| MC87 | C4 | 5-10 | 676 - 878 CE (95.4%) |
| MC87 | C4 | 10-15 | 686 - 885 CE (95.4%) |
| MC87 | C5 | 10-15 | 662 - 774 CE (95.4%) |
| MC87 | C5 | 35-40 | 680 - 880 CE (95.4%) |
| GALB | RH4 | 15-20 | 2876 - 2585 BCE (95.4%) |
| GALB | C3 | 5-10 | 666 - 822 CE (95.4%) |
| GALB | C7 | 40-45 | 7739 - 7588 BCE (95.4%) |
| GALB | C10 | 10-15 | 1410 - 1460 CE (95.4%) |
| GALB | C10 | 15-20 | 1326 - 1438 CE (95.4%) |
| GALB | C10 | 20-25 | 1421 - 1490 CE (95.4%) |
| GALB | C10 | 25-30 | 1410 - 1455 CE (95.4%) |
| GALB | C11 | 20-25 | 2894 - 2638 BCE (95.4%) |
| GALB | C12 | 20-25 | 706 - 891 CE (95.4%) |
| GALB | C12 | 30-35 | 690 - 887 CE (95.4%) |
| NOUR | C11 | 20-25 | 442 - 640 CE (95.4%) |
| NOUR | C11 | 25-30 | 430 - 584 CE (95.4%) |
| NOUR | C11 | 35-40 | 660 - 776 CE (95.4%) |
| NOUR | C12 | 30-35 | 4230 - 3982 BCE (95.4%) |
| NOUR | C13 | 10-15 | 1396 - 1446 CE (95.4%) |
| NOUR | C13 | 15-20 | 1296 - 1398 CE (95.4%) |
| NOUR | C15 | 5-10 | 1306 - 1420 CE (95.4%) |
| NOUR | C15 | 15-20 | 648 - 774 CE (95.4%) |
| NOUR | C15 | 20-25 | 664 - 774 CE (95.4%) |
| NOUR | C17 | 10-15 | 772 - 976 CE (95.4%) |
