# Supplementary material for: Landscape-scale spatial variations of pre-Columbian anthropogenic disturbances at three ring ditch sites in French Guiana
Source: PLoS One. 2024 Sep 26;19(9):e0298714. doi: 10.1371/journal.pone.0298714 (PMC11426519; doi:10.1371/journal.pone.0298714)
Supplement: S3 Table — Model 1: Soil property ~ Soil type (with soil type: ferralsols, hydromorphic gleysols). Model 2: Soil property ~ Site + Localization + Depth (with site: MC87, NOUR, GALB; location: ring ditch enclosure and ditch, ring ditch hilltops and slopes, adjacent hills). * p-value < 5%, ** p-value < 1%. (DOCX) [file pone.0298714.s011.docx]

**S3 Table. Generalized linear model analyses of soil physicochemical properties.** Model 1: Soil property ~ Soil type (with soil type: ferralsols, hydromorphic gleysols). Model 2 : Soil property ~ Site + Localization + Depth (with site: MC87, NOUR, GALB ; location: ring ditch enclosure and ditch, ring ditch hilltops and slopes, adjacent hills). * p-value < 5%, ** p-value < 1%.

| **Soil Property** | | **Model 1** | **Model 2**  **(on terra-firme soils only)** | | |
| --- | --- | --- | --- | --- | --- |
|  |  | **Soil type effect (p-value)** | **Study site effect (p-value)** | **Landscape localization (p-value)** | **Depth p-val** |
| PSD | Clay | 2.56×10^-56^ ** | 8.28×10^-31^ ** | 4.18×10^-3^ ** | 1.82×10^-08^ ** |
|  | Fine silt | 0.87 | 0.08 | 4.28×10^-4^ ** | 1.45×10^-03^ ** |
|  | Coarse silt | 6.31×10^-07^ ** | 0.07 | 0.10 | 0.02 * |
|  | Total silt | 2.66×10^-3^ ** | 0.10 | 2.57×10^-04^ ** | 6.42×10^-03^ ** |
|  | Fine sand | 3.71×10^-58^ ** | 9.76×10^-12^ ** | 1.49×10^-06^ ** | 1.16×10^-07^ ** |
|  | Coarse sand | 2.31×10^-45^ ** | 1.43×10^-78^ ** | 0.77 | 5.24×10^-07^ ** |
|  | Total Sand | 7.90×10^-64^ ** | 1.19×10^-68^ ** | 0.06 | 8.31×10^-10^ ** |
| Chemistry | pH | 1.46×10^-10^ ** | 0.71 | 0.02 * | 7.31×10^-13^ ** |
|  | Corg | 4.74×10^-21^ ** | 1.97×10^-07^ ** | 4.53×10^-4^ ** | 1.49×10^-51^ ** |
|  | Ntot | 6.16×10^-11^ ** | 0.23 | 1.86×10^-4^ ** | 1.98×10^-100^ ** |
|  | P | 0.35 | 4.51×10^-2^ * | 0.73 | 0.93 |
|  | K | 5.79×10^-07^ ** | 0.70 | 0.43 | 1.10×10^-44^ ** |
|  | Mg | 8.09×10^-23^ ** | 3.84×10^-24^ ** | 4.71×10^-09^ ** | 2.94×10^-13^ ** |
|  | Ca | 1.30×10^-32^ ** | 0.73 | 1.75×10^-3^ ** | 1.16×10^-09^ ** |
|  | Na | 1.30×10^-14^ ** | 0.10 | 0.92 | 2.77×10^-13^ ** |
|  | Al | 0.29 | 3.86×10^-16^ ** | 0.02 * | 2.67×10^-17^ ** |
