# Supplementary material for: Landscape-scale spatial variations of pre-Columbian anthropogenic disturbances at three ring ditch sites in French Guiana
Source: PLoS One. 2024 Sep 26;19(9):e0298714. doi: 10.1371/journal.pone.0298714 (PMC11426519; doi:10.1371/journal.pone.0298714)
Supplement: S1 File — (DOCX) [file pone.0298714.s012.docx]

**S1 File. Construction of the composite index of anthropogenic disturbance.**

A composite index is a metric that combines multiple variables into a single variable. The construction of such an index relies both on previous knowledge and expectations and empirical variations in a given dataset. The present index was based on the following soil properties (called *P* in the following, where $P_{pit,d}$ refers to the value of the *P* variable in a given pit at a given depth):

- Presence/absence of ceramic shards (weight =1)
- Presence/absence of macrocharcoals (weight =0.5)
- Presence/absence of soots (weight =0.5)
- Organic carbon content, Corg (weight =1)
- Total nitrogen content, Ntot (weight =1)
- Magnesium content, Mg (weight =1)
- Calcium content, Ca (weight =1)
- Aluminum content, Al (weight =1)
- Munsell color value (weight =1)
- Munsell color chroma (weight =1)

Phosphorus (P) and potassium (K) were not included in the present index, as these properties did not vary significantly between landscape-scale localizations in our study. Microcharcoal counts were not included in the index as their abundance was estimated in a reduced number of pits only. Because macrocharcoals and soots provide complementary information, these variables are weighted by a factor of 0.5 (cf. step 3).

Step 1. Variable Direction

Because some variables are expected to decrease while anthropogenic disturbance increases (e.g., color variables: the lower the value, the darker the color and the lower the chroma, the stronger the color), these variables are first transformed to reverse their direction of variation by multiplying each value by ‘-1’ : ${-P}_{pit,d}=P_{pit,d}\times(-1)$

Step 2. Variable Scaling

Data are normalized into a range [0-1] through min-max scaling. This step standardizes variables with different variation ranges (e.g., variables expressed in different units), while preserving the original distribution of the variables.

${P'}_{pit,d}=\frac{P_{pit,d}- min(P)}{max(P) - min(P)}$, where $min(P)=min{\{P_{pit,d}\}}_{pit=1;d=1}^{Npit;Nd}$and $max(P)=max{\{P_{pit,d}\}}_{pit=1;d=1}^{Npit;Nd}$

Step3. Index Computation

For each pit and depth,

$I_{pit,d}=\sum_{p=1}^{Np} ({P'}_{pit,d}\times W_{p})$, where $W_{p}$ is the weight of each *P* variable:

$$W_{p} =\{1,0.5,0.5,1,1,1,1,1,1,1\}$$

Then, for each pit,

$$I_{pit}=\frac{\sum_{p=1}^{Np} (I_{pit,d})}{Nd}$$
