# Supplementary material for: Landscape-scale spatial variations of pre-Columbian anthropogenic disturbances at three ring ditch sites in French Guiana
Source: PLoS One. 2024 Sep 26;19(9):e0298714. doi: 10.1371/journal.pone.0298714 (PMC11426519; doi:10.1371/journal.pone.0298714)
Supplement: S2 File — (PDF) [file pone.0298714.s013.pdf]

Inclusivity in global research

PLOS' policy on inclusivity in global research aims to improve transparency in the reporting of research performed outside of researchers' own country or community and ensures that PLOS publications reporting global research adhere to high standards for research ethics and authorship. Authors of relevant research articles may be asked to complete the questionnaire below, which outlines ethical, cultural, and scientific considerations specific to inclusivity in global research. This questionnaire may be requested when researchers have travelled to a different country to conduct research, if research uses samples collected in another country, research with Indigenous populations or their lands, or if research is on cultural artefacts. Researchers travelling to another country solely to use laboratory equipment will not normally be required to complete the questionnaire. However, the questionnaire can be requested at the journal's discretion for any submission – if you have been requested to complete this questionnaire by the PLOS journal you submitted to, please do so.

Please complete the questionnaire below and include this as a Supporting Information file with your manuscript. Note that if your paper is accepted for publication, this checklist will be published with your article in the supporting information files. Please ensure that you reference the checklist in the main body of your manuscript. We suggest adding a subsection 'Inclusivity in global research' to your Methods section and adding the following sentence: "Additional information regarding the ethical, cultural, and scientific considerations specific to inclusivity in global research is included in the Supporting Information (SX Checklist)"

The questions have been designed to be applicable to a wide range of study types, and there are subsections for both human subjects research and non-human subjects research. If any of the questions are not relevant to your research please mark them as "N/A" as appropriate.

Ethical considerations, permits and authorship

This section is applicable to all research types.

Provide details as to who granted permissions and/or consent for the study to take place in the Methods section of your manuscript. This should include the names of **all** ethics boards, governmental organizations, community leaders or other bodies that provided approval for the study. If individuals provided approval refer to these people by their role or title but do not list their name(s).

Reported on page number: 8

If there were any deviations from the study protocol after approval was obtained please provide details of these changes in the Methods section of your manuscript.

Not applicable (no deviation from the study protocol).

Did this study involve local collaborators that are residents of the country where the research was conducted or members of the community studied? If you do not have any authors from said communities, please provide an explanation for this below.

All authors are researchers or research associates affiliated to local institutions. MT, KM, JE and LB are members of the French National Institute for Sustainable Development in Cayenne (IRD Cayenne, French Guiana). MM is member of the Institute for Archaeological and Preventive Research (INRAP) in French Guiana. All authors are or were residents of French Guiana when the study was carried out.
We did not study modern local communities and did not access to traditional knowledge from local communities. Today, there is no local communities living in the study sites.

Everyone listed as an author should meet PLOS' criteria for authorship and all individuals who meet these criteria should be included in the author byline, rather than the acknowledgements. For further information please see the journal's Authorship Policy.

Human subjects research (e.g. health research, medical research, cross-cultural psychology)

Did you obtain written informed consent from a representative of the local community or region before the research took place? How did you establish who speaks for the community? Details of written informed consent obtained from study participants should be reported separately in the Methods section of your manuscript.

Not applicable (non-human research).

How did members of the local community provide input on the aims of the research investigation, its methodology, and its anticipated outcome(s)?

Not applicable (non-human research).

When engaging with the local community, how did you ensure that the informed consent documents and other materials could be understood by local stakeholders?

Not applicable (non-human research).

Will the findings of the research be made available in an understandable format to stakeholders in the community where the study was conducted (e.g. via a presentation, summary report, copies of publications, etc.)? Please provide details of how this will be achieved.

Not applicable (non-human research).

Non-human subjects research using specimens/animals collected as part of the study, or those housed in archival collections. Examples include archaeology, paleontology, botany and zoology.

Did the permission you obtained from a local authority to perform the study include an agreement on access to outputs and benefit sharing? This may include procedures to enable fair distribution of the benefits and resources arising from the research performed. Please include any details of Prior Informed Consent and Benefit Sharing Agreements obtained. These may be required by field-specific regulations, for example the Convention on Biological Diversity (CBD) and the associated Nagoya Protocol.

As part of the DOPAMICS project, access to genetic resources was approved by the Ministry of Ecological transition (decision of March 02, 2021) and by the CBD Access and Benefit Sharing Clearing House (Internationally Recognized Certificate of Compliance no ABSCH-IRCC-FR-254777-1).

DOPAMICS team members are strongly involved in benefit sharing with local students and citizens. We welcome and train students from the University of French Guiana and from the Agricultural High School in French Guiana. We organized, in collaboration with the Amazonian National Park, the nature festival in Saül (the nearest village to Mont Galbao) : on this occasion, we introduced local schoolchildren and residents to the scientific methods involved in the study through “field-to-lab” activities in the form of an outdoor excursion followed by a “mini-laboratory” (https://www.ird.fr/la-fete-de-la-nature-saul-guyane-francaise-avec-lequipe-dopamics ; https://parc-amazonien-guyane.fr/fr/actualites/de-belles-fetes-de-la-nature-dans-le-sud-guyane). We also participate in local conferences to make our finding available and understandable for the general public.

All data acquired are available in Open Access.

Our research is not subject to any commercial valorization or monetary profit.

If the material used in your study was imported, please A) provide the year it was imported and B) indicate whether permits were obtained to import/export the materials used, C) provide details of any permits obtained. If this information is not available, please indicate this.

We imported soil material from French Guiana to the European territory to carry out lab analyses (subcontracted). In accordance with the Commission Delegated Regulation (EU) 2019/829 of 14 March 2019, the import of non-European soil samples into the European Union territory for physicochemical analyses was authorized by an Official Authorization Letter of 27 July 2023.

If you used archival specimens, please state how the material used in your study was acquired by the institute it is held in and provide details of any permits obtained for the original excavations/ sample collection. If this information is not available, please indicate this.

Not applicable (no use of archival specimen).

How was the potential cultural significance of the materials collected in your study to local communities considered in your research design? Were Indigenous peoples and/or local researchers and institutions involved with archaeological excavations/collection of specimens? If so, please provide a description of their involvement.

Thematic archaeological survey was authorized by local authorities (prefectoral decision no. 2022-73 of 12 September 2022). Shards contained in the soil samples consisted of fragments that are not culturally identifiable. In the event of the discovery of major or complete archaeological material, we were instructed to leave it *in situ* and to transmit the geographical coordinates to the archaeological service of French Guiana but, unfortunately, this was not the case.
As mentioned in the section "Ethical considerations", the study involved researchers and research associates from local institutions. No local communities were involved in fieldwork.

If your manuscript includes photographs of human remains please indicate whether authors obtained permission from descendants or affiliated cultural communities to do so.

Not applicable (no photographs of human remains)
